# High-resolution structure determination using high-throughput electron cryo-tomography

**DOI:** 10.1107/S2059798322005010

**Published:** 2022-06-07

**Authors:** Hsuan-Fu Liu, Ye Zhou, Alberto Bartesaghi

**Affiliations:** a Department of Biochemistry, Duke University School of Medicine, Durham, NC 27708, USA; b Department of Computer Science, Duke University, Durham, NC 27708, USA; c Department of Electrical and Computer Engineering, Duke University, Durham, NC 27708, USA

**Keywords:** electron cryo-tomography, sub-volume averaging, *in situ* structure determination, beam-image shift tomography, constrained single-particle tomography

## Abstract

Tomographic reconstruction of frozen-hydrated specimens followed by extraction and averaging of sub-tomograms has successfully been used to determine the structure of macromolecules in their native environment at resolutions that are high enough to reveal molecular level interactions. The low throughput characteristic of tomographic data acquisition combined with the complex data-analysis pipeline that is required to obtain high-resolution maps, however, has limited the applicability of this technique to favorable samples or to resolutions that are too low to provide useful mechanistic information. Recently, beam image-shift electron cryo-tomography (BISECT), a strategy to significantly accelerate the acquisition of tilt series without sacrificing image quality, was introduced. The ability to produce thousands of high-quality tilt series during a single microscope session, however, introduces significant bottlenecks in the downstream data analysis, which has so far relied on specialized pipelines. Here, recent advances in accurate estimation of the contrast transfer function and self-tuning exposure-weighting routines that contribute to improving the resolution and streamlining the structure-determination process using sub-volume averaging are reviewed. Ultimately, the combination of automated data-driven techniques for image analysis together with high-throughput strategies for tilt-series acquisition will pave the way for tomography to become the technique of choice for *in situ* structure determination.

## Abstract 2

In this article, it is shown that high-throughput strategies for tomographic data acquisition combined with unsupervised techniques for image analysis provide the foundation for closing the resolution gap between the high-resolution strategies used to study molecular assemblies reconstituted *in vitro* and techniques for *in situ* structure determination.

## 1. Introduction

**Figure 1:**
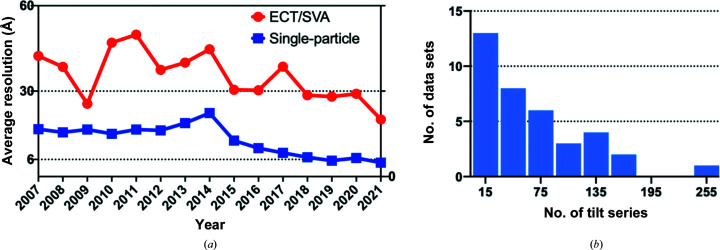
Resolution trends for SPA and ECT/SVA and the average number of tilt series deposited per data set in the EMPIAR database. (*a*) Historical average resolutions obtained from the EMDB reported for structures determined using ECT/SVA (red) and SPA (blue). (*b*) Histogram of the total number of tilt series deposited per data set (mean = 50), reflecting the low throughput characteristic of ECT.

Single-particle analysis (SPA) is routinely used to determine the structure of macromolecular complexes at high resolution by imaging randomly oriented but otherwise identical macromolecules distributed within a thin layer of vitrified ice (Bendory *et al.*, 2020 ▸; van Heel *et al.*, 2000 ▸; Singer & Sigworth, 2020 ▸). In contrast, electron cryo-tomography (ECT) allows the study of macromolecular complexes within their crowded biological context by producing three-dimensional tomograms of individual pleomorphic environments reconstructed from a series of projections recorded while tilting the microscope stage (Bartesaghi & Subramaniam, 2009 ▸; Walz *et al.*, 1997 ▸; Asano *et al.*, 2016 ▸; Zhang, 2019 ▸). Repeating instances of biological entities can be identified, extracted and combined using sub-volume averaging (SVA) to remove the noise and obtain high-resolution structures (Bartesaghi *et al.*, 2008 ▸; Sanchez *et al.*, 2020 ▸; Liu *et al.*, 2008 ▸). The resolution of maps determined by SVA is generally lower than those produced by SPA (Fig. 1 ▸ *a*), in part due to the technical difficulties of imaging native specimens and the lack of mature analysis tools that can effectively extract high-resolution information from noisy data (Zhang, 2019 ▸; Bartesaghi *et al.*, 2005 ▸; Frank *et al.*, 2012 ▸; Kuybeda *et al.*, 2013 ▸; Meyerson *et al.*, 2011 ▸; Zanetti *et al.*, 2009 ▸). In addition, the low-throughput characteristic of ECT data collection has severely limited the size of the data sets that can be acquired and used for SVA, with the mean number of tilt series per data set deposited in the Electron Microscopy Pilot Image Archive (EMPIAR) database being only 50 (Fig. 1 ▸ *b*). Despite these challenges, several high-resolution structures of naturally concentrated samples have been obtained using this technique (Schur *et al.*, 2016 ▸; Tegunov *et al.*, 2021 ▸). The first subnanometre-resolution structure determined by ECT was obtained using the constrained single-particle tomography (CSPT) data-processing paradigm (Bartesaghi *et al.*, 2012 ▸), where the idea of working directly with the raw 2D projections extracted from the tilt series and performing an SPA-like reconstruction was first introduced. This hybrid strategy for data analysis provides an effective framework to process high-resolution tilt series data by leveraging the use of established principles in SPA image reconstruction and refinement (Walz *et al.*, 1997 ▸; Bartesaghi *et al.*, 2015 ▸; Glaeser, 2019 ▸).

In SPA data collection, navigating cryo-EM grids using beam image-shift (BIS) is a rapid way to increase the number of targets acquired per stage movement without sacrificing resolution (Cheng *et al.*, 2018 ▸; Wu *et al.*, 2019 ▸). Our recently proposed beam-image shift electron cryo-tomography (BISECT) strategy extends the BIS principle to ECT, making it possible to reduce the acquisition time by an order of magnitude without sacrificing resolution (Bouvette *et al.*, 2021 ▸). This dramatic increase in throughput, however, poses unique challenges for the downstream data-analysis pipeline that consists of multiple processing steps and requires significant amounts of expert user intervention. For example, robust estimation of the contrast transfer function (CTF), which is critical for achieving high resolution, is very challenging to perform accurately due to the low contrast of tomographic projections. In addition, how best to account for the lower quality of tilted projections and the effects of radiation damage that occur during exposure is still poorly understood. To address these challenges, we recently introduced robust routines for unsupervised tilted CTF estimation and self-tuning exposure weighting (Bouvette *et al.*, 2021 ▸). Combined with high-speed acquisition using BISECT, these advances will bring this technology a step closer to becoming a high-throughput tool for *in situ* structure determination.

## 2. Methods

### 2.1. Parallel acquisition of tilt series using beam-image shift (BIS)

**Figure 2:**
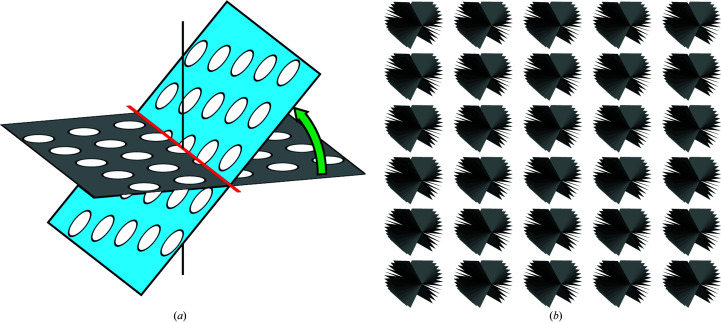
Parallel acquisition of tilt series for high-resolution tomography using BISECT. (*a*) Unlike previous approaches for tomographic data collection that acquire tilt series in a serial manner, BISECT is the first example of the simultaneous acquisition of tilt series, which is capable of collecting data at high magnification without sacrificing resolution. (*b*) Parallel acquisition of tilt series using a 5 × 5 beam-image shift pattern produces 25 simultaneous tilt series in about 2–3 min per tilt series using a Titan Krios microscope and a K3 camera.

Achieving subnanometre resolution using ECT combined with SVA requires the acquisition of multiple projections from the same region of interest (ROI) taken from different angles and with a well defined defocus. To ensure precise targeting, tracking areas are often used to estimate deviations induced from mechanically tilting the sample so that they can be compensated for during acquisition. To successfully implement BISECT, the movement of each ROI in all three dimensions needs to be considered. As the stage is tilted, targets that do not lie along the tilt axis will move away from the focus plane (*Z* axis) and inwards towards the tilt axis. Using the same tracking principle to re-center on the target area, the new positions of each ROI can be estimated from their previous locations. Using this procedure, it is possible to set up image-shift patterns following a regular holey lattice or arbitrary patterns where each ROI is manually chosen (Fig. 2 ▸). The actual number of target areas determines the effective speedup factor that can be achieved, which in practice ranges from 1 to 25 depending on the type of grid, the type of sample and the number of areas suitable for imaging present within the maximum allowable BIS distance (Bouvette *et al.*, 2021 ▸).

To satisfy the sampling-rate requirements of high-resolution imaging, pixel sizes of 1.5 Å or smaller need to be used. At these magnifications, however, the field of view is rather small on the detector and the precision of the stage is in the same order of magnitude. Without performing corrections between tilts, the targeted area would drift outside the field of view, especially when using a dose-symmetric scheme in which the stage is subject to large rotations. BISECT uses a two-step tracking procedure to achieve a targeting precision of better than 10 nm over the entire course of the tilt series, allowing the routine acquisition of tilt series at high magnification.

The most promising approaches for *in situ* structure determination involve the preparation of frozen-hydrated sub­cellular slices either by serial sectioning (Schwartz *et al.*, 2003 ▸; Bharat *et al.*, 2018 ▸) or by focused ion beam (FIB) milling (Zachs *et al.*, 2020 ▸; Schaffer *et al.*, 2015 ▸). The target areas identified using fluorescent markers, however, are often too large to be sampled at atomic resolution using current detectors. Although sampling could be extended by acquiring consecutive tilt series by mechanical displacement of the specimen, this approach would be too slow to be practical. The ability of BISECT to image an arbitrary pattern of areas in a radius of approximately 8–10 µm allows the parallel acquisition of several tilt series spanning the size of an average mammalian cell, thus effectively providing a path for overcoming this limitation. Moreover, when compared with serial acquisition by mechanical displacement, BISECT requires a significantly lower number of tracking images, thus reducing the overall radiation damage that is likely to destabilize the specimen, making it suitable for high-throughput acquisition of thinned subcellular slices at the magnifications that are needed for high-resolution imaging.

### 2.2. Robust estimation of CTF parameters from raw tomographic tilt series

**Figure 3:**
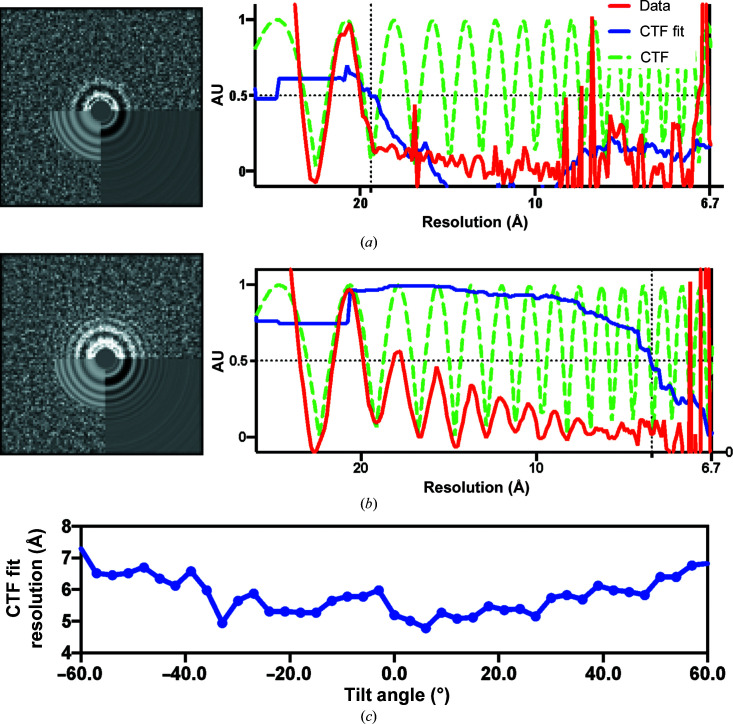
Robust CTF estimation from tomographic tilted projections obtained by leveraging tilt-geometry parameters determined during tilt series alignment. (*a*) Left: a 2D power spectrum from a 60° tilt projection and CTF estimation results obtained using *CTFFIND*4 (Rohou & Grigorieff, 2015 ▸), which is designed to work on untitled images, shows only the first few rings of the CTF. Right: radial average of the 2D power spectrum (red line), theoretical CTF curve (green dashed line) and corresponding CTF-fit curve measuring the similarity between the red and green curves (blue line). (*b*) Corresponding power spectrum and CTF estimation profiles for the image shown in (*a*) using the tilted CTF estimation strategy that uses information from tilt series alignment to successfully recover the Thon ring pattern extending to higher resolution. (*c*) CTF-fit resolution cutoff as a function of the tilt angle. Unlike strategies that rely on averaging consecutive tilts to improve the SNR (Mastronarde & Held, 2017 ▸), this constrained approach produces accurate per-tilt defocus estimates throughout the ±60° tilt range.

While semi-supervised and fully unsupervised strategies for tilt series alignment and tomographic reconstruction are used routinely in ECT (Mastronarde & Held, 2017 ▸), other downstream steps such as CTF estimation and high-resolution SVA still require significant amounts of user input. Accurate defocus estimation, for example, is critical for correctly performing deconvolution of the CTF during 3D reconstruction and is a prerequisite for achieving high resolution. Small errors arising from imprecisions in the detection of zero-crossings can lead to signal attenuation, particularly at higher resolutions. While strategies for fully astigmatic defocus estimation and correction are used routinely in SPA, their use in ECT has been challenging for two main reasons: (i) the lower contrast of dose-fractionated tilted projections produces weak Thon rings, making defocus estimation unreliable, and (ii) the defocus gradient induced by tilting requires the use of specialized strategies for CTF estimation that can properly account for the change in defocus (Fig. 3 ▸ *a*). To overcome these challenges, previous attempts have made simplifying assumptions, such as imposing conditions of eucentricity of the tilt series (Fernández *et al.*, 2006 ▸), assuming negligible or constant astigmatism per tilt series (Chen *et al.*, 2019 ▸), using small subsets of projections in order to improve the signal-to-noise ratio (SNR) (Xiong *et al.*, 2009 ▸) or using data from strips with minimal defocus variation which can lead to inaccurate estimates, especially at high tilt angles (Turoňová *et al.*, 2017 ▸). Meanwhile, strategies for collecting projections from tilted specimens were introduced in SPA to address problems of preferred particle orientation that lead to lower or anisotropic resolution reconstructions (Tan *et al.*, 2017 ▸). This led to the development of programs for tilted defocus determination that can track the change in defocus as a function of the tilt angle (Su, 2019 ▸; Zhang, 2016 ▸; Mindell & Grigorieff, 2003 ▸). While this strategy is not directly applicable to tilt series images due to the lower doses used in tomography, since the tilt axis and tilt angle for each projection are determined precisely during tilt series alignment (Mastronarde & Held, 2017 ▸), these parameters can directly be used as input to existing SPA routines that are used for tilted CTF estimation. This approach allows the reliable estimation of defocus and astigmatism for all images in a tilt series (even at high tilt angles), despite the low contrast characteristic of ECT (Figs. 3 ▸ *b* and 3 ▸ *c*).

### 2.3. Self-tuning exposure weighting improves ECT/SVA map resolution

In the CSPT refinement paradigm (Bartesaghi *et al.*, 2012 ▸), 3D reconstruction and refinement is carried out using 2D particle projections extracted directly from the raw tilt series. To maximize the recovery of high-resolution information, we recently implemented data-driven exposure-weighting routines similar to those used to improve the resolution of SPA reconstructions (Bartesaghi *et al.*, 2018 ▸; Zhou *et al.*, 2020 ▸). Exposure weights for each tilt angle are estimated based on the average similarity measured between raw particle projections and the most recent 3D reconstruction (Grant *et al.*, 2018 ▸). The newly derived weights automatically reduce the contribution of high-tilt images or projections taken later in the tilt series, allowing the more efficient extraction of high-resolution information from the raw tilt series data. Since this strategy is purely data-driven, it requires no knowledge of the tilting scheme or the electron exposure used during data collection, which can be difficult to determine accurately in practice. Compared with approaches that compensate for this effect by applying fixed critical exposure curves (Grant & Grigorieff, 2015 ▸), the use of self-tuning strategies has already been shown to outperform these filtering strategies both in SPA (Bartesaghi *et al.*, 2018 ▸) and CET (Tegunov *et al.*, 2021 ▸; Bouvette *et al.*, 2021 ▸) imaging.

## 3. Results

**Figure 4:**
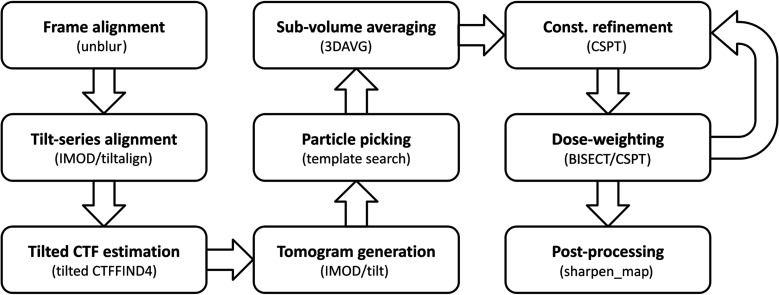
End-to-end protocol for high-resolution structure determination using constrained single-particle tomography. The sequence of data-processing steps and software programs used to convert raw tilt series into high-resolution 3D structures using the original 2D tomographic particle projections is shown.

**Figure 5:**
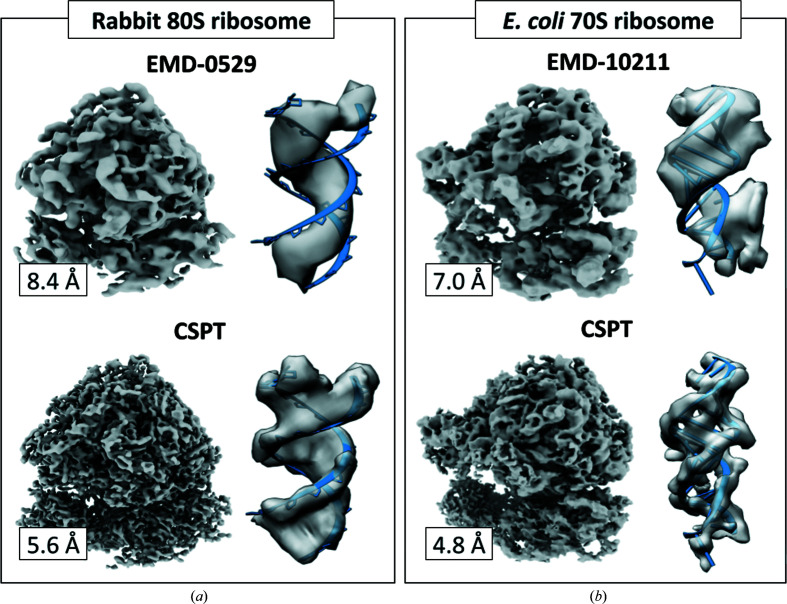
Data-driven CSPT refinement improves ECT/SVA map resolution. (*a*) Reconstructions obtained using tilt series of rabbit 80S ribosomes (EMPIAR-10064). The original 8.4 Å resolution map (EMDB entry EMD-0529; top) and the 5.6 Å resolution map (EMDB entry EMD-23357) obtained using CSPT (bottom) are shown. (*b*) Reconstruction of *E. coli* 70S ribosomes (EMPIAR-10304). The 7.0 Å resolution map (EMDB entry EMD-10211) and the 4.8 Å resolution map (EMDB entry EMD-23358) obtained using CSPT (bottom) are shown (Bouvette *et al.*, 2021 ▸).

To show the combined effects of these advances in image processing on map resolution, we executed the end-to-end workflow illustrated in Fig. 4 ▸ to reprocess tilt series from mammalian 80S ribosomes (EMPIAR-10064; Khoshouei *et al.*, 2017 ▸) and *Escherichia coli* 70S ribosomes (EMPIAR-10304; Eisenstein *et al.*, 2019 ▸). The resolutions obtained compared favorably with those obtained by other packages using the same raw data (EMD-10211 and EMD-10840) and resulted in improvements of ∼3 and ∼2 Å, respectively (Fig. 5 ▸).

**Figure 6:**
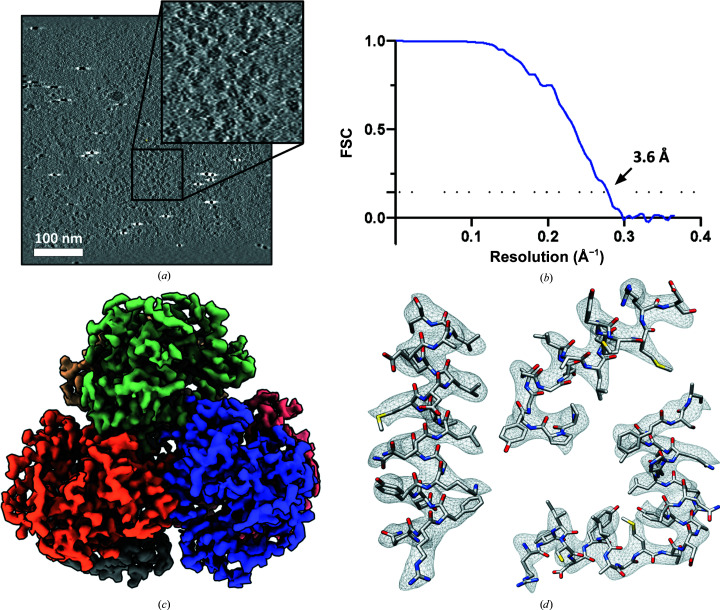
High-resolution structure of a 300 kDa complex obtained using BISECT and CSPT. (*a*) 50 nm thick tomographic slice showing individual particles (inset). The scale bar indicates 100 nm. (*b*) Fourier shell correlation (FSC) between half-maps (3.6 Å resolution according to the 0.143 cutoff criterion). (*c*) dNTPase map (EMDB entry EMD-23356). (*d*) Visualization of density for side chains.

To determine the applicability of these techniques to more challenging targets of smaller molecular weight, we imaged a monodisperse sample of *Vibrio cholerae* dNTPase, an HD-domain family protein and a homolog of SAMHD1 and *E. coli* dGTP triphosphohydrolase. dNTPase is a homohexamer with *D*3 symmetry and a total molecular weight of ∼300 kDa. Using BISECT, we collected a total of 275 high-magnification tilt series using a K2 camera at a speed of 5 min per tilt series. We estimated the tilted CTF for each projection independently and selected 64 tilt series based on the absence of ice contaminants and the best estimated CTF resolution. From the reconstructed tomograms, 34 000 particles were extracted and subjected to sub-volume averaging followed by CSPT refinement, resulting in a 3.6 Å resolution map where density for side chains can be visualized (Bouvette *et al.*, 2021 ▸; Fig. 6 ▸).

## 4. Conclusion

While the number of SPA structures deposited in the Electron Microscopy Data Bank (EMDB; Velankar *et al.*, 2016 ▸) has increased steadily over the last few years, progress in CET has been slower due to the technical challenges involved in imaging tilted biological specimens using low electron doses. Only recently, the advent of platforms for high-speed data collection and high-resolution data processing have significantly improved the technical capability of CET, paving the way for the routine visualization of targets imaged *in situ* at near-atomic resolution. Imposition of the constraints of the tilt geometry during refinement and reconstruction allows the accurate alignment of particle projections and proper estimation of the CTF while overcoming the low SNR of tilted projections and minimizing overfitting. Future developments in data processing will result in additional improvements in resolution that could soon allow the application of these methods to even lower molecular weight targets (<300 kDa). Overall, the advent of technological advances in CET will allow this technique to become an effective strategy to routinely study protein complexes at near-atomic resolution within the functional context of the cell. Importantly, at these resolutions the visualization of individual side chains will greatly facilitate the placement of atomic models into cryo-EM maps (Brown *et al.*, 2015 ▸; Afonine *et al.*, 2018 ▸), resulting in better quality structures. Ultimately, these methods will help to close the resolution gap between the high-resolution strategies used to study molecular assemblies reconstituted *in vitro*, such as X-ray crystallography and SPA, and techniques for *in situ* structure determination, such as CET/SVA.
